# Changeability of Disease

**Published:** 1858-08

**Authors:** J. W. Dowsing

**Affiliations:** Atlanta, Ga.


ATLANTA

Medical and Surgical Journal.

Vol. III.] AUGUST, 1858. [No. XII.

### ORIGINAL COMMUNICATIONS.

### ARTICLE I.

*Changeability of Disease.* By Dr. J. W. Dowsing, Atlanta, Ga.

From whatever point of view we survey the vast empire of nature, changeability, or mutation, would seem to be one of the results of her natural laws. So far as we can observe, this principle is common, except in degree, alike to the animate and inanimate, the organic and inorganic, worlds. And when we take an extensive cosmical view, we find that it pervades not only our terrestial globe, but have reason to infer that it attends the celestial bodies, in their revolving migrations, to the remotest borders of the universe. If we regard the earth in its retrospect, we find that its history has been one continued series of successive changes. Each one of her geological systems is a volume, and every stratum a chapter upon the revolutions and transmutations that have taken place in her body. Every era has had its peculiar geological features and aspects; and from the mysterious depths of Paleontology, we glean that each period has had its peculiar plants and animals — gradually rising in the scale of perfection, through the successive periods, from the Graywacke to the Historic—rising in the vegetable kingdom, from the fucoides and flowerless plants of the Graywacke, to the graceful palms and magnolias of the Historic; and in the animal kingdom, from the mollusca, and radiata genera of the former, to the different varieties of man of the latter. Nor do the varied aspects, which the face of the earth every where present, bespeak a less constant change than the history of the past reveals. Two of the elements of ancient philosophers, earth and water, are at constant war with each other, and each contending for the supremacy. At one point the ocean is encroaching upon the land; and at another place, plutonic forces are rearing the land, in the form of islands, above the surface of the great deep. Thus is the aspect of the earth's surface continually changing.

In the vegetable kingdom, this law obtrudes itself forcibly upon us. So susceptible are plants to this principle, that cultivation has the power of so transforming their nature, as to render some of the most uninteresting species of the primeval valleys at once the most beautiful and delightful, as well as the most useful of our gardens and orchards. From the hard and acrid nature of the native apple of the forest, has decended the delicious, mellow, and palatable fruit of cultivated orchards; while the soft and delicious peach has its origin from a shrub, noted for producing one of the most poisonous fruits found in the primeval forests of the East.

If, in the vegetable world, this law is found to prevail, it is still more strikingly developed in the animal. Among the beasts of the field, the birds of the air, or the inhabitants of the waters, it is constantly manifesting itself. At the head of the animal creation comes man,

> "At first the infant
> Mewling and puking in the nurse's arms;
> And then the whining school-boy with his satchel,
> And shining morning face, creeping like a snail,
> Unwillingly to school;" and the bold and courageous cur of the northern latitudes, becomes the degenerate and hairless animal of Turkey and Nicaragua; while the floundering Porwiggle of to-day becomes the full formed, leaping frog of to-morrow. And who has not observed with mingled emotions of wonder and admiration, the great transformation to which many tribes of insects are subjected? To-day we mark with disgust the loathsome and crawling caterpiller; to-morrow we scrutinize closely the dormant aurelia, only to behold with delight, after a brief period, the gaudy and fluttering butterfly. But wonderful as these changes may seem, they are not more remarkable than the transmutation from life to death. In the dead body, so soon as the vital force, or power of cohesion has taken its flight, all things hasten to unite with each other, according to their particular laws. Combinations which have had a temporary existence, and in which the art of man may detect and separate substances, which he would seek in vain, in the interior of the earth, and in the fluid ocean of air and water, are broken down, and return to their natural elements. Inert matter, says the great modern master of science, Baron Humbolt, "animated awhile by vital force, passes through an innumerable diversity of forms, and perhaps in the same substance which once enshrined the spirit of a Pythagoras, a poor worm may have enjoyed a momentary existence."

It would, indeed, seem that the vital force itself was subject to change, or rather vascillating in the uniformity of its strength. In embryotic animals, and in the germ of plants, it is weak, and consequently is easily destroyed, but gradually becoming stronger, and its powers of assimilation more powerful until it reaches maturity, when it as uniformly grows weaker, and becomes less capable of containing or holding inorganic matter in subjugation; no longer able to assimilate, it yields the substance in which it has temporarily enshrined itself, to the laws of the inorganic world. Seeing, then, that the very elements of man's existence are constantly assuming new forms, and seeking new affinities, we naturally conclude, *a priori*, that the various maladies to which he is subject, would be influenced by the same immutable laws; and that his disorders must, of necessity, present a diversity of features and aspects; and this is in accordance with the history of disease. It would be as great an anomaly in the practice of medicine, to find two cases of disease with symptoms in all respects similar to each other, as it would be in nature to find two human faces, with features entirely identical. In proof of this, we have but to refer to common observation, and the history of the past. That the cold plague, which was once the terror and scourge of the Middle Ages, was a form of Asiatic Cholera, there can be no doubt; and that it was materially different in many of its features, is as certainly true. Nor can there be but little doubt, that the famous pestilence that once scourged proud Athens, and the destructive plague that depopulated London in 1666, were closely allied to the same germ of disease.

But for evidence of the changeability of disease, we need not recur to semimythical annals of ancient history, nor to the less fabulous pages of modern record. We have but to examine and investigate with ordinary accumen, the diseases that fall under our own observation. Our lots are cast in an age, in a country, and under circumstances eminently calculated for the observation of this principle in disease, and it can not have failed to have attracted the attention of the most superficial observer. Besides, the many natural causes, such as constitutional changes of the atmosphere, geological formations and electrical phenomena, which tend to modify and vary the aspect of disease, there are many social conditions, which act as powerful auxiliaries to the promotion of that end. Man is not stationary in his habits, but is ever changing his physical, political and social condition; and as in the progress of time, these changes in the human race must continue to increase and become more complex, in the same ratio will the intricacy of his disorders increase. The pioneers of a new country, provided it be fertile, well watered and climate favorble, pays the penalty of encroaching upon the forest and uncultivated lands, by suffering frequent attacks of autumnal, intermittent and remittent fevers; while the inhabitants of old and cultivated countries suffer from gout, neuralgia, mania, consumption and scrofula. He that has yielded, in any considerable degree, to the migratory character of our people, cannot tail to have noticed the gradual change of disease, in our newly settled States. First comes autumnal, intermittent and remittent fevers—from the mildest form of intermittent to its most malignant grade. As the country becomes older and more settled, these diseases gradually give way, presenting in the mean time, every phase of their respective types, until they are finally supplanted by dysenteries and continued fevers. Even in my native State, (Mississippi,) which is comparatively a young State, districts in which but a few years since, no type of fever was known, save intermittents and remittants, are now superseded by these diseases. Or, if, indeed, a case of the old fashioned autumnal, or remittent fever occur, it requires the greatest caution, and closest vigilance in the treatment, to prevent it from running into the continued type. Our endemical diseases will, in time, leave our country, and will perhaps, be known to posterity only in history. Already the most formidable of the group has, in a manner disappeared, and but for the faithful and accurate history given of it by one of our worthy brothers, would, in all probability, have been known to future generations, only through the tradition of their ancesters.

It must be admitted that there has been of late years, a natural tendency in most of our diseases to assume a typhoid action; yet the past two has been more exempt than the preceding ten. How far this condition is dependent upon natural causes, such as meteorological changes, cultivation, &c., and how far upon injudicious treatment, remains for future experience and observation to determine. That there are many cases of artificial typhoid, brought about by injudicious treatment, chiefly by the excessive use of purgatives, I have no doubt. There is too much indiscriminate purging in the treatment of our fevers. It is true that many diseases require purgatives, and many others tolerate them, without rapid prostration; but as a general thing, they are more abused in domestic practice, and I am sorry to admit, by some practitioners, than any other class of medicines in the whole materia medica.

Periods may occur in which most diseases bear the use of purgatives with impunity; but this does not argue their indiscriminate use. The dogma of Hamilton and Cooke had its origin in one of these periods; but it has been persevered in by their disciples and followers, without regard to the constitutional changes of disease, to a fearful and mischievous extent. Many of them regard the liver as *the* offending organ in almost every malady, and according to their favorite doctrine, commence torturing it with blue mass, Cook's pills, calomel, and in fact the various purgatives of mercury, and continue it for an incredible length of time. Some physicians of this school seem to consider their patients Promethian-livered; and as Jupiter bound Prometheus of old with chains, and sent an eagle to prey on his liver, which grew every night as much as it had lost in the day, so these modern Jupiters, in medicine, confine their patient, and send a vulture, in the form of mercury, to prey on their livers at night. The chief difference in the comparison arises in the liver of Prometheus being inconsumable, while that of the modern patient is highly vulnerable.

Mortifying as may be the acknowledgment, I am forced to admit, that I have seen physicians, who stood high in the profession in the community, prescribe a dose of mercury almost every night, for weeks in succession, when nothing in their condition required its administration. How, then, can we expect otherwise, than that this valuable agent should fall into disrepute with the community, and become at once the *opprobium medicorum*, when the community see, daily, its deleterious effects from such rash empiricism? But let me not be understood as being in any way opposed to the judicious use of this valuable medical agent. Nothing is farther from my purpose, than to attempt, in any degree, to disparage it as a remedy. It is only to its abuse, its too common administration, which has brought it into such odium with the community, as to render it difficult of administration, without disguising it, that I object. So much opposed are many persons to using it, even when their condition requires it, that the practitioner is sometimes tempted to resort to the most artful stratagems in order to induce them to take it. That this state of affairs has been brought about by its too common and indiscriminate use, there can be no doubt.

It is, then, an axiom in medicine, that diseases are not only constantly assuming new features and aspects, but, also, new forms and types. In our intermittent, remittent, and continued fevers, the changeability of disease is strikingly manifested; since they are continually alternating in type, and running into each other. Intermittents frequently run into remittents, and sometimes *vice versa;* and often has the practitioner seen, both to his regret and chagrin, remittents assume the continued variety, in despite of, seemingly, the most skillful treatment.

In some of our low Southern States, bordering upon the Atlantic and Gulf coast, especially in Louisiana, about New Orleans, the summer and autumnal diseases have a decided tendency to crisis by hemorrhage; and according to Dr. Fenner, this crisis makes yellow fever; since "it forms the true characteristic difference between the high degrees of summer and autumnal fevers in the city and country, and must depend on locality and attendant circumstances. During the healthiest years it predominates over all other types; but during the sicklier years, in the country it runs into remittent, bilious and congestive; whilst in the city it runs into yellow fever. Dr. Harrison testifies, that he has often observed malignant intermittents to precede the outbreak of yellow fever."

In autumnal diseases, I have occasionally been not a little puzzled in making out a diagnosis—the symptoms so obscurely marked, and so blended with each other, that it has been difficult to decide whether the case before me was one of bilious, intermittent, or remittent; and I have sometimes been not a little mortified to find that it was neither, but a genuine specimen of continued fever. Of this difficulty of diagnosis, Dr. Fenner remarks with peculiar force: "Physicians may say what they please about being able to distinguish a case of yellow fever as soon as they examine it; we do not believe it possible, according to their ideas. Rarely does a summer pass, in which we do not hear of some intelligent and experienced practitioner being perfectly astonished at seeing what he had pronounced a case of intermittent, or remittent bilious fever, terminate in black vomit or other hemorrhage."

But, thanks to a bountiful Providence, it matters little what phases our febrile diseases may wear, or what types they may assume, we have a remedy with which, as a general thing, we do not fear to meet them, even though they present as many forms and shapes as Proteus himself. The sulphate of quinine, as a remedy in fevers, whether billious, intermittent, remittent or continued, has never been too much valued, nor too highly extolled. The sedative powers of large doses of quinine in fevers, were first proclaimed to the profession by Dr. Fenner, and have been subsequently endorsed by the army surgeons, and the physicians of the southern and western States, who have constantly urged its claims as a sedative, in the various Medical Journals in the United States, for the last fifteen or twenty years. These views are gradually extending to the north, and when properly and impartially tested there, must certainly produce a revolution in therapeutics. Dr. Fenner is a sanguine advocate of large doses of quinine in yellow fever, and urges the excellence of the abortive treatment, of that disease, with the utmost confidence. Dr. Dundas, of Liverpool, recommended the same treatment in the continued fevers of England. The wonderful powers of sulphate of quinine are forcibly set forth in a short essay, by the lamented Prof. Harrison, of New Orleans, in the second volume of the New Orleans Medical and Surgical Journal, and subsequently by several other eminent practitioners of the same city. In the continued fever of certain localities in Alabama and Mississippi, previous to an epidemic which prevailed during the autumn of 1854, and spring and summer of 1855, my experience and testimony is in behalf of quinine, as a most powerful remedial agent. When given in large doses, say ten or twelve grains, every two or three hours, and continued until thirty or forty grains are taken, it seldom fails to be followed by the happiest results. I know that there are those who entertain a contrary opinion, but, as yet, I have never known any injury to result from prescribing it, in however large doses, or however long continued; but in the great majority of instances, a marked amelioration of all the febrile symptoms has immediately followed its administration. And even in those cases in which the febrile symptoms were not abated, the patient was subject to no inconvenience, save, perhaps, some unpleasant ringing in the ears, and a slight temporary derangement of the head. It is true, by the use of this remedy, there is some pecuniary loss, which should not be disregarded, particularly if the patient is poor; but when we use quinine efficiently, in any form of idiopathic fever, both the physician and patient have the consolation to know that they have tried one of the most reliable and efficacious febrifuges known to the medical world. I would not be understood as assuming the ground that quinine is capable of arresting all cases of typhoid fever; on the other hand, I believe it is not. In many instances I have administered full doses, in which it seemed to exert no controlling influence. But in the majority of these cases, the disease was remarkably insiduous in its approach—the patient often being indisposed with slight head-ache and fevers, for a week or more before I was consulted. Had full doses of the medicine been given at the very out-set of the disease, the result would, perhaps, have been different, but as it was, the weight of evidence was against its use. And if I had never seen the happy effects of quinine, in typhoid fever, in other seasons, localities and climates, I should have been disposed to condemn it, as a remedy in this form of fever. But its failure served only to impress me with the great medical truth, that disease, viewed from different points, presents new aspects, and requires new modes of treatment. Many a Medical Knight has shivered his lance against the shield of his antagonist, when, perhaps, an interchange of position would have convinced each other, that both were right and both were wrong. And had some of our talented physicians in Atlanta practiced medicine in New Orleans, and Dr. Fenner in Atlanta, they might, perhaps, approximate each others views on the points, more closely than they do at present.

In the Panama fever, which I should consider nothing more nor less than a continued form of remittent fever of the tropics, full doses of quinine, it is asserted, usually either cuts short the disease, or prevents the accession or increase of the more formidable symptoms. Indeed, a friend of mine, who remained some time in Panama, says, that "quinine, given in full doses in the incipient stage of the disorder, in good constitutions, seems to act as a specific, and at once frees the patient from all symptoms of fever.

Such constitutions, or conditions of the atmosphere as produce influenza, measles, scarlatina, whooping-cough, cholera, dengue, and other wide spread maladies, which occasionally visit us, undoubtedly have a decided modifying influence over endemic and sporadic diseases. The prevalence of our fevers, dysenteries, etc., is frequently promoted, and modified in their nature, by what may be termed a pestilential or abnormal condition of the atmosphere. When the spread of these complaints is extensive, and their malignancy and devastation usually formidable, I apprehend that they are always thus promoted—their peculiar poison being aided in its action by some malign condition of the atmosphere.

These changes, or constitutions, of the atmosphere come and recede, and have appeared and disappeared, ever since the earliest authentic record of our race, without revealing to mortal eye, even their existence, except through their effect on health and vitality. An opinion on them could, therefore, be purely conjectural; consequently, I will not offer one, but leave their disclosure to the observing, intelligent and faithful students of nature, and the sage teaching of time.

But novel as the idea may be deemed, I believe there are irregular periodic constitutions of the atmosphere, in which the *vis conservatrix naturæ* and healthful action of the economy is depressed, and a general tendency prevails in the system, to take on disease of a malign or depressing character; so, also, there are periods which the converse is true, and diseases of a highly inflammatory type prevail. These periods are not regular in their appearance, but like comets, appear and disappear at uncertain and irregular intervals. Sometime, for a series of years, pleurisy, pneumonia, phrenitis, inflammatory rheumatism, and other diseases of a high inflammatory type, seem mostly to prevail, which require and bear depletion and contra-stimulants to their fullest extent. But, perhaps, in a brief space of time, these conditions of things are changed. The maladies which existed in the same locality, a short time since, have altered these types; and now, instead of bearing depletives, their use must be sedulously avoided; while carminatives, anodynes and stimulants, must be early and energetically administered in their place.

Nor can there be any reasonable doubt, that this *changeability* in disease, is the prime and efficient cause of the origination of the various dogmas and systems of medicines, which have at different times been thrust upon a credulous world. It is well known that Thompson projected his ultra stimulant theory at a time when spotted or continued fever raged in his vicinity to an alarming extent. Being a low grade of fever, it required stimulants from the outset. Luckily he fell upon the practice of administering his favorite hobby, lobelia, and the various preparations of pepper, with the happiest success, while his cotemporaries pursued their accustomed practice of purging and depletion, with the worst consequences. Inflated with temporary success, he at once imagined that he had found in these few agents the universal remedy for the cure of all diseases, and consequently proclaimed his favorite theory to an all credulous and ever confiding world. The result is known to all. But in a few short years the condition of things were again changed. In the same locality, in which a short time previously he was hailed with so much applause, the continued fevers had disappeared, and were replaced by pleurisies, and other diseases of a highly inflammatory type. He again resorted to his favorite remedies; but now his treatment was as fatal, as it was successful before. On the other hand, the regular physicians treated the diseases on general antiphlogistic principles with marked success. The consequence was, that his theory immediately exploded, and he himself fell into disrepute, in the sphere of his own immediate influence. His opinions were no longer respected at home, but he had already propagated his doctrine to the world, which is scarcely yet fully dissipated; for, as Jefferson once quaintly remarked, "error will travel a thousand miles before truth can get his boots on." But truth will soon be round, and the doctrines of Thompsonianism will, ere long, be known only as one of the novel absurdities of the past. Yet it should teach our profession the wholesome lesson, that we should ever keep a vigilant watch on the changes of disease; discriminate in the treatment of maladies; and cease to persevere in the use of a given class of remedies, simply for the sake of theory. The "science of medicine is a great unroofed temple," and much yet remains to be accomplished. Let us, then, not discard a valuable suggestion, or a good remedy, because it comes recommended by an objectionable system, since it is the duty of every judicious physician to select from all systems that which may prove valuable or useful.

Homœopathy is another beautiful system of refined nonsense, which must run its course—rise, flourish, decline, and ultimately be consigned to the tomb of oblivion, like its antetypes, the abracadabra and royal touch. But from it we may be taught several important lessons. It demonstrates to us in a striking manner, the credulity of mankind. The generality of man wonder and marvel at a matter, in proportion as they are ignorant of its laws; and the more astounding and unreasonable the dogma of the designing imposter, the more prompt are they to believe, and bow in low humility to his shrine. Only a few practical hits, no matter how grossly misrepresented, are all that are necessary to give circulation to the vainest chimera that ever emenated from the imagination of the most fanatical visionary. It is vain to expostulate with them about the impropriety of fostering such nonsense. They virtually tell us that all our ratiocinations must be reversed, and the infinitesimal fraction of an atom must be regarded as possessing more power than the whole. If the preposterous system of Hahneman be true, the boasted philosophy of Bacon, Newton, Franklin, and a number of other luminaries of the scientific constellation, must be regarded as dreamy visions, which have been unjustly considered philosophy by an ignorant and benighted world. More than fifty, yea, sixty, years have elapsed since the doctrine of Homœopathy was promulgated. It has subsequently been pronounced to be utterly false and unworthy of confidence, after having been thoroughly tested by some of the best physicians of the age.

M. Andral, one of the first practical observers in medicine that Europe has ever produced, declares that he experimented on several individuals in *health*, with belladona, cinchona, aconite, &c., and in no single instance have the promises of Homœopathy been verified. This distinguished physician has also tested the merit of this system upon more than one hundred cases of *sick*, and though he made these trials with such boasted articles as aconite, conchona, belladonna, etc., he could not perceive that they produced any effect. Yet Andral procured his medicines from a Homœopathic pharmaceutist, and scrupulously observed the special regimen, such as the great father of the system orders.

Few enlightened and honorable physicians of the present day, have been proselyted to this new doctrine. Prof. Henderson, of Edenburg, the most prominent one, was expelled several years since, with disgrace, from the medical society of that city, in consequence of it; and observation proves, that it has been seldom adopted, either in Europe or in this country, except by men of inferior standing, and who could only get into practice by the aid of *novelties*, not to say duplicity and humbuggery. A writer in the New Orleans Medical and Surgical Journal justly remarked: "It is gravely charged upon such as have left the regular profession to follow this new light, that they practice the grossest imposition upon their employers by availing themselves of whatever popularity may attach to the new system, while they pursue mainly the old. Such conduct merits, and must, sooner or later, obtain the scorn and contempt of all honorable men."

But homeopathy, after all, may ultimately result favorably to the cause of science. Besides convincing man of the credulity of his race; the duplicity and transcendental pretensions of its author and his followers; the ridiculous absurdities of its theory; and the inert efficacy of its practice, it further teaches us the important truth, that the great majority of the complaints of mankind do not necessarily endanger life, and may get well under almost any plan of treatment; or, if you will, without any course of treatment at all. It will be seen, then, that the chief secret and beauty of the system is a reliance on the *vis medicatrix naturæ.* This is the chief oasis in their desert system; and it would be well, perhaps, for us to profit more generally from it.

In more than one instance have I known patients to be pronounced hopeless, and left to die, recover by virtue of this mysterious principle of our nature. But this principle is not always adequate to the emergency, and it remains for the judicious physician to decide when to aid the natural healing and conservative powers of nature by medicinal agents, and when to desist in their use.

Next comes the late system of Hydropathy, with its long train of amphibia, alternately swathed in wet sheets, and sweating to discharge the *peccant humors* from the blood through the skin. That these aquatic followers of Prietsnitz can and have treated, efficiently, many diseases by the application of water, I do not pretend to question; but I am not yet prepared to admit that water is the universal panacea for all diseases. But there is nothing new in the treatment of diseases by the local application of water. It has been used as a remedial agent in medicine, ever since the day of the Epidausian god; and every judicious physician will continue to use it whenever symptoms arise that indicate its employment. But to use it indiscriminately, and attempt to treat all disorders with it, is as utopian in theory, as it is rash and empirical in practice.

We have already stated on a previous page, that changeability of disease was probably the chief cause of the existence of the various dogmas and systems in medicine. There has ever been a veil of mystery thrown around the temple of medicine, and whatever is wonderful in theory, or novel in practice, has always been eagerly grasped by the credulous and ignorant. From unseen natural causes, changes in social condition, &c., diseases are constantly assuming new forms and types, and the remedies which proved efficient in the diseases of yesterday, may prove futile and nugatory in those of to-morrow. This naturally begets doubt and distrust in the minds of the masses, and they are at once ready to hail, with enthusiasm, anything in the shape of medicine, or doctrine, that bears with it the impress of mystery and novelty. The unprincipled and designing imposter, seeing this state of things, and conscious of the unbounded credulity of mankind, prepares some worthless secret nostrum, or proclaims a *theory*, nor cares what may be the result, so that his purse is well lined with gold. Or the wild visionary, blinded with infatuation, may promulgate a doctrine impelled by the frenzied belief that he would thereby ameliorate the condition of man, and cause his name to be emblazoned to a grateful world as a benefactor of his race. Thus it will be seen, that he may be criminal in the matter, and deserve obloquy; or *innocent*, and merit the commiseration of his fellows.

Besides the meteorological changes, habits of life, food, regimen, geological position, etc., will form other important modifying causes in the peculiarities of disease. As our country grows older, and becomes more densely populated, the luxury of life will continue to increase, and the habits of the people become more artificial and complicated. From these causes disease will continue to change. Remittent and intermittent fevers, which are now so common, will diminish in frequency, and fevers of an irritative character will prevail, and ultimately take their place. And these irritative or continued fevers will be greatly modified in their nature and as pect, by constitutional and hereditary diseases; for the presumption is, that the latter will continue to increase. It is true, that some may, possibly, by the aid of medical science, be eradicated, but the probability is, that the great majority of them will occur more frequently, and become more complicated and difficult to manage, as time progresses.

Diseases of a tuburculous and scrofulous character will continue to be the scourges of our race, and will increase in virulence, as they are transmitted from one generation to another. And as we descend the great stream of life, they will undoubtedly prevail as much as now, and will, in all probability, assume new forms, which, if seen now, would be strange and anomalous. With each successive generation we may look for an increased proportion of those who have a tuburculous or scrofulous taint. In future ages, when large cities shall have sprung up in our now sparcely settled country, with a crowded population, filthy and ill-fed inhabitants, and ill-ventilated residences, the increase of cachectic diseases will be greatly favored; and as these conditions continue to multiply, diseases of a scrofulous character will be found to abound in almost every form.

The syphylitic will continue to increase, and in its remote effects, as transmitted from parent to child, it may become so common as to be interwoven with almost every form of morbid derangement, and thus become a prolific source of chronic disease. "The older a people becomes," says Dr. Burr, of New York, "the more syphylitic complications will be found." Generations yet to come will have to pay the penalty of their father's transgressions in the form of many kinds of anomalous diseases. "To a certain extent we may, at the present time, see some of the consequences of syphylitic taint; and when chronic derangements presented themselves in a form inexplicable and strange, my suspicions would not be excited, and I should search for evidences of venerial contamination, either in the patient before me, or in some of his immediate ancestors. The cause of these complications cannot, in all probability, be ckecked, but will continue to multiply until the consequence will form no inconsiderable portion of the future chronic diseases of this people."—Burr.

The neurosis will also become more common. Lunacy, and mental affections generally, will become a prominent disease. The character of our Government, morals and politics, will operate as so many causes to increase the number of cases of mental derangement among the people, and will also tend to give new forms, varieties and phases, to their manifestations.

Gout and other diseases, connected with personal habits, practices and pursuits, though minor in themselves, will continue to increase, and will exercise an important modifying influence over other forms of diseases. Goitre and cretinism, in some localities, will become common, and will no doubt, in the course of time, give such a variety of type to other diseases in their locality, as to require some peculiarity of treatment.

I have already assumed the ground that changeability is not only found in disease, but is one of the natural results of the laws of the universe. Man himself is constantly changing; as a natural consequence the disorders to which he is subject must necessarily assume new forms and aspects. At certain periods of life, one temperament passes into another, as the result of the natural changes which take place in the process of the growth of the human body. This subject is so interesting, that it presents a theme for an extended paper, which cannot be entered upon here.

Like every thing else in nature, the diseases of mankind are never stationary. Of the system of man, the same is true. From birth to death it presents an uninterrupted succession of changes; and death itself is but another change. Nor are the mutations of disease less strikingly manifested.

Such, in brief, are some of the views I have entertained respecting the *changeability of disease*, and the causes that diversify it. If they are correct, time will ratify them, and further observation fill up the outline I have so imperfectly sketched. In that case, like all other truths, they may prove useful. They do not rest on visionary conceit, but have a tangible basis that can be approached and examined. But should they be found erroneous, they will only share the fate of the thousand perishable fancies that have preceded them, and no one will regret their subversion less than myself.

